# Brain alterations of regional homogeneity, degree centrality, and functional connectivity in vulnerable carotid plaque patients with neither clinical symptoms nor routine MRI lesions: A resting-state fMRI study

**DOI:** 10.3389/fnins.2022.937245

**Published:** 2022-08-05

**Authors:** Qian Wang, Wu Xing, Lirong Ouyang, Lang Li, Hong Jin, Shuai Yang

**Affiliations:** ^1^Department of Radiology, Xiangya Hospital, Central South University, Changsha, China; ^2^National Clinical Research Center for Geriatric Disorders, Xiangya Hospital, Central South University, Changsha, China

**Keywords:** resting-state fMRI, carotid plaque, vulnerable plaque, regional homogeneity, degree centrality

## Abstract

**Aims:**

Based on resting-state functional MRI (fMRI), we preliminarily explored brain alterations in asymptomatic patients with vulnerable carotid plaques, but carotid stenosis was < 50%.

**Methods:**

A total of 58 asymptomatic patients with vulnerable carotid plaques (stenosis <50%) and 38 healthy controls were recruited. Between-group differences in regional homogeneity (ReHo), degree centrality (DC), and functional connectivity (FC) were analyzed. Correlation analysis was performed between the ReHo or DC values in altered brain regions as well as voxel-wise abnormal FC and scores on neuropsychiatric scales, serum interleukin-6 (IL-6), and C-reactive protein (CRP).

**Results:**

Both ReHo and DC values on the left superior occipital gyrus (SOG.L) of the asymptomatic vulnerable carotid plaque group reduced, regardless of plaque location (left, right, or bilateral). Functional connections weakened between the SOG.L and right lingual gyrus (LING.R)/right inferior occipital gyrus (IOG.R), right middle frontal gyrus (MFG.R)/orbital part of superior frontal gyrus (ORBsup.R)/orbital part of middle frontal gyrus (ORBmid.R), left precentral gyrus (PreCG.L)/postcentral gyrus (PoCG.L), left supplementary motor area (SMA.L), right paracentral lobule (PCL.R), left precuneus (PCUN.L), and right postcentral gyrus (PoCG.R)/PCL.R. In ReHo-altered brain regions, ReHo values were positively correlated with Hamilton Rating Scale for Depression (HAMD) scores, and the setting region of abnormal ReHo as seed points, voxel-wise FC between the SOG.L and PreCG.L was negatively correlated with CRP.

**Conclusions:**

Cerebral alterations of neuronal synchronization, activity, and connectivity properties in the asymptomatic vulnerable carotid plaque group were independent of the laterality of vulnerable carotid plaques. Significant relation between ReHo values on the SOG.L and HAMD indicated that even when there were neither clinical symptoms nor lesions on routine MRI, brain function might have changed already at an early stage of carotid atherosclerosis. Inflammation might play a role in linking vulnerable carotid plaques and changes of resting-state functional connectivity.

## Introduction

Atherosclerosis, a chronic inflammatory disease (Wolf and Ley, 2019), leads to cardiovascular and cerebrovascular diseases as its main clinical consequences. The vulnerability of atherosclerotic plaques is one of the key characteristics to evaluate the severity of the cardiocerebrovascular disease and the risk of plaque rupture. At the same time, atherosclerosis has been proved to be related to cognitive dysfunction and dementia (Cortes-Canteli and Iadecola, 2020; Xie et al., 2020; Gardner et al., 2021). Identifying atherosclerosis at the subclinical stage is an important strategy to improve the effect of the primary prevention of cardiovascular disease (Ahmadi et al., 2019; Planas-Ballvé et al., 2019; Kristensen et al., 2020). However, there are still many difficulties in the early sign of central nervous system diseases (including stroke and vascular cognitive impairment) related to atherosclerosis.

Moderate to severe carotid stenosis caused by carotid atherosclerotic plaques could lead to alterations in brain structures or brain functions, and long-term clinical outcomes of patients who were referred for duplex ultrasonography and found to have <50% stenosis were not benign (Palamuthusingam et al., 2011). Some research demonstrated that vulnerable carotid plaques might relate with silent infarction and white matter hyperintensity in the brain (Freilinger et al., 2012; Moroni et al., 2016). Recently, some studies have used resting-state functional MRI (fMRI) to study the relationship between carotid stenosis and cognitive impairment. Cheng et al. (2012) performed resting-state fMRI on patients with asymptomatic severe carotid artery stenosis caused by atherosclerosis and found that the functional connectivity (FC) within and between the left and right hemispheres of the brain was significantly lower than that in the control group. Huang et al. (2018) also found that FC in the ipsilateral cerebral hemisphere of patients with carotid artery stenosis before carotid stenting decreased. After carotid artery stenting, some FC changes gradually returned to normal. The greater the degree of FC abnormality (whether low connectivity or high connectivity), the worse the cognitive performance, especially in memory and executive functions. He et al. (2021) found that the brain FC of patients with left asymptomatic carotid stenosis (≥70%) decreased in the left and right inferior frontal gyri, temporal lobe, left cingulate gyrus, and hippocampus and was related to short-term memory impairment.

The aforementioned studies partially confirmed the association between carotid stenosis and brain damage or alterations; however, there are still some problems. First, after the carotid artery stenosis is relieved or recanalized, the recovery of cognitive function is not ideal (Plessers et al., 2014). Therefore, whether hemodynamic changes are the only pathological mechanism causing cognitive dysfunction in patients with carotid artery stenosis is unknown, and other possible pathological mechanisms need to be explored. A study of Tawakol et al. (2017) directly confirmed that arterial inflammation is related to regional brain activity in the amygdala and showed that amygdala activity independently and reliably predicted the occurrence of cardiovascular disease. Inflammation, as one of the characteristics of vulnerable plaques, may also become a potential pathological mechanism to understand the correlation between atherosclerosis and brain and cognitive functions. Second, most of the studies were carried out in the population with more than 50% of carotid artery stenosis, when is in a relatively late stage. It is necessary to explore whether the population at a relatively early stage of carotid atherosclerotic plaques (defined as the presence of carotid plaques but carotid stenosis smaller than 50% in this study) has early brain activity changes. Third, the cause of carotid artery stenosis or the type of carotid plaques has not been discussed in the previous studies.

Based on the problems mentioned earlier, our research used ReHo and degree centrality (DC) through resting-state fMRI to observe changes in regional brain functional activity and connectivity properties in asymptomatic volunteers with vulnerable carotid plaques but carotid stenosis smaller than 50%, and explore the potential relationship between vulnerable carotid plaques and brain alterations and the other possible pathological mechanisms in addition to hemodynamic changes.

## Materials and methods

### Study subjects

This study was approved by the ethics committee of Xiangya Hospital of Central South University. All volunteers were recruited from the National Stroke Screening and Intervention Project, who were at high risk of stroke (participants older than 40 years with ≥3 of traditional vascular risk factors). Considering the safety of conducting this study, we excluded subjects older than 75 years. We recruited volunteers with vulnerable carotid plaques in bifurcations of common carotid arteries (CCAs) or internal carotid arteries (ICAs) but carotid stenosis smaller than 50%, and normal controls from January 2017 to December 2019. Vulnerable carotid plaques were diagnosed by color Doppler ultrasonography of the carotid artery. The inclusion criteria were as follows: (1) aged from 40 to 75 years; (2) Han (the predominant ethnic group in China); (3) right-handed; (4) no heart disease or mental illness; (5) no mental or psychophysiological trauma; (6) no evidence of cardiogenic embolism; (7) no drug, medicine, or alcohol dependence; (8) white matter hyperintensities Fazekas < 2; (9) no other craniocerebral lesions; and (10) no cranial artery abnormalities. The exclusion criteria were as follows: (1) clinical history of carotid endarterectomy and carotid artery stenting; (2) atrial fibrillation; (3) drug and alcohol addicts; (4) severe hepatorenal disease; (5) pregnancy; (6) left subclavian artery plaque; and (7) artifact on MR sequences. Finally, 58 volunteers with vulnerable carotid plaques but carotid stenosis smaller than 50% and 38 normal controls without carotid plaques were ultimately included. Among the diagnosed subjects, 17 subjects had vulnerable plaques in the left carotid artery, 15 subjects had vulnerable plaques in the right carotid artery, and 26 subjects had vulnerable plaques in bilateral carotid arteries. We recorded demographic information and risk factors for stroke (including age, gender, race, education level, alcohol intake, smoking history, obesity, family history, past history, pressure, and lifestyle) through a questionnaire; administered the Mini-Mental State Examination (MMSE), Hamilton Rating Scale for Anxiety (HAMA), and Hamilton Rating Scale for Depression (HAMD); conducted resting-state fMRI; and collected 10 ml blood samples to measure serum interleukin-6 (IL-6) and C-reactive protein (CRP).

### Carotid ultrasound and MRI data acquisition

Examination of carotid plaques was performed in both the right and left bifurcations of CCAs and ICAs by the same experienced radiologist using an ultrasound scanner (5-MHz linear array transducer; iU22, Philips Ultrasound, Bothell, WA). Clinically, vulnerable carotid plaques diagnosed by color Doppler ultrasonography is described as isoechoic, hypoechoic, or mixed echogenicity plaques with irregular or ulcerated surfaces (Gray-Weale et al., 1988; Hermus et al., 2010).

MRI data were acquired using a GE 3.0 MRI scanner (Signa HDx; General Electric Healthcare, Milwaukee, WI, United States) with a typical 8-channel head coil. Imaging series included axial 3D T1-weighted imaging (T1WI), axial T2-weighted imaging (T2WI), axial T2-weighted fluid-attenuated inversion recovery (T2-FLAIR), MR angiography (MRA), and rs-fMRI sequences. The axial 3D brain volume (3D-BRAVO) sequence consisted of 188 slices per volume, and the parameters were as follows: repetition time (TR) = 7.792 ms, echo time (TE) = 2.984 ms, inversion time (TI) = 800 ms, field of view (FOV) = 256 × 256 mm^2^, flip angle (FA) = 7°, thickness = 1 mm, and spatial resolution = 1 × 1 × 1 mm^3^. Rs-fMRI data were obtained from a gradient-echo echo-planar imaging sequence with the following scanning parameters: 180 time points, 32 slices, TR = 2,000 ms, TE = 30 ms, FOV = 220 × 220 mm^2^, FA = 90°, thickness = 4 mm, matrix = 64 × 64, and spatial resolution = 3.438 × 3.438 × 4.000 mm^3^.

### Data analysis

Rs-fMRI data were analyzed in Data Processing Assistant for Resting-State fMRI Advanced Edition (DPARSFA, http://rfmri.org) and Statistical Parametric Mapping 12 (SPM12, http://www.fil.ion.ucl.ac.uk/spm/software/).

#### Preprocessing

The preprocessing of resting-state fMRI data was conducted in DPARSFA and consisted of the following steps: 1 format conversion; 2 removal of the first 10 time points; 3 slice timing correction; 4 realignment for head motion correction, and according to the head motion correction curve, the participants with a displacement >2.5 mm or an angular rotation >2.5% were excluded (among 96 cases, three cases were excluded: two cases in the asymptomatic vulnerable plaque group and one case in the normal control group); 5 regression of nuisance including head motion parameters (24 parameters, Friston 24-parameter model; Friston et al., 1996; Yan et al., 2013), brain white matter intensity, and cerebrospinal fluid intensity; and 6 spatial normalization and resampling, using a Diffeomorphic Anatomical Registration Through Exponentiated Lie Algebra (DARTEL) toolbox, data were normalized to the Montreal Neurological Institute (MNI) standard space and resampled to a voxel size of 3 mm × 3 mm × 3 mm; and 7 bandpass filtering (0.01–0.08 Hz). Statistics were conducted on gray matter masks defined within the groups.

#### ReHo analysis

First, we calculated the similarity of the time series between each voxel and its 26 neighboring voxels to obtain Kendall's coefficient of concordance (KCC). Then, the KCC value of each voxel was divided by the mean value of all voxel KCCs in the whole brain to obtain the standardized ReHo value (Zang et al., 2004). Finally, the ReHo map was smoothed with an 8-mm full width at half maximum (FWHM) isotropic Gaussian kernel.

#### DC analysis

Voxel-based DC means the number of connections between a node or brain region and all nodes or brain regions. Based on nodes or brain regions, DC can reflect centrality in the brain network and describe the importance of nodes or brain regions in the whole brain. DC reflects brain function completely, rapidly, and efficiently (Buckner et al., 2009; Zuo et al., 2012). The DC map was smoothed with an isotropic 8 mm FWHM Gaussian kernel.

#### Voxel-wise functional connectivity analysis

After smoothing preprocessed data with an 8 mm FWHM isotropic Gaussian kernel, we calculated the FC maps. We selected brain regions with altered ReHo and DC values in the asymptomatic vulnerable plaque group comparing to the control group as masks, extracted the mean time series of the brain regions, and analyzed resting-state FC by using the voxel-wise method, which means comparing the time series of these brain regions to those of every voxel in the whole brain. Finally, we converted the correlation coefficients resulting from FC into z values by Fisher z-transformation.

### Statistical analysis

SPM12 and xjView (http://www.alivelearn.net/xjview) were used to identify the MNI coordinates of anatomical brain regions showing statistically significant differences between groups. The independent samples *t*-test was performed to compare the ReHo and DC values of the asymptomatic vulnerable plaque group and normal control group (*p* < 0.05, family-wise error (FWE) correction). To estimate the influence of position laterality of vulnerable carotid plaques (17 left, 15 right, and 26 bilateral), within-subjects analysis of variance (ANOVA) was performed, followed by a correction for multiple comparisons (Bonferroni test). Meanwhile, to verify the reliability of our results, brain regions with abnormal ReHo and DC values were extracted to perform multiple comparisons among the normal control group and the left, right, and bilateral asymptomatic vulnerable plaque groups, followed by a correction for multiple comparisons (Bonferroni test). FC analysis was conducted using DPARSF. The FC results were compared between the two groups using an independent samples *t*-test in statistical parametric mapping, or SPM (*p* < 0.05, FWE correction). Considering the possible influence that age might have on our results from a clinical perspective, we conducted linear regression with age and ReHo, and age and DC, separately. SPSS software was used to conduct Pearson correlation analysis and partial correlation analysis to analyze the ReHo and DC values and the MMSE, HAMA, and HAMD scores (with age and education level as covariates). SPSS software was used to conduct Pearson correlation analysis to analyze the ReHo and DC values and serum IL-6 and CRP. SPSS software was also used to conduct Pearson correlation analysis and partial correlation analysis (with age and education level as covariates) to analyze voxel-wise abnormal FC and neuropsychiatric scales, serum IL-6, and CRP; *p* < 0.05 was considered statistically significant.

## Results

### Demographic and clinical data

For the asymptomatic vulnerable plaque group and normal control group, there were no statistically significant differences in gender, age composition, systolic blood pressure, diastolic blood pressure, blood sugar, serum triglycerides, total cholesterol, high-density lipoprotein cholesterol (HDL-C), low-density lipoprotein cholesterol (LDL-C), or homocysteine (see Table 1).

**Table 1:** Demographic and clinical data.

	**Asymptomatic vulnerable plaques group (*n* = 58)**	**Normal controls group** **(*n* = 38)**	***p*** **-Value**
Age (years old)	61.19 ± 6.85	58.26 ± 7.53	0.05
Male, No./total No. (%)	26.00 (44.83%)	17.00 (44.74%)	0.34
Systolic blood pressure (mmHg)	144.25 ± 18.93	136.32 ± 15.65	0.13
Diastolic blood pressure (mmHg)	88.92 ± 10.86	85.59 ± 12.74	0.35
Blood sugar (mmol/L)	6.20 ± 3.02	5.31 ± 1.20	0.16
Serum triglycerides (mmol/L)	2.29 ± 1.27	2.61 ± 2.44	0.49
Cholesterol (mmol/L)	5.57 ± 1.21	5.70 ± 0.83	0.64
HDL-C(mmol/L)	1.32 ± 0.44	1.37 ± 0.46	0.66
LDL-C(mmol/L)	3.76 ± 0.95	3.41 ± 0.69	0.11
Homocysteine (μ mol/L)	13.28 ± 8.49	13.18 ± 3.48	0.96

### ReHo indicator

**Figure 1:**
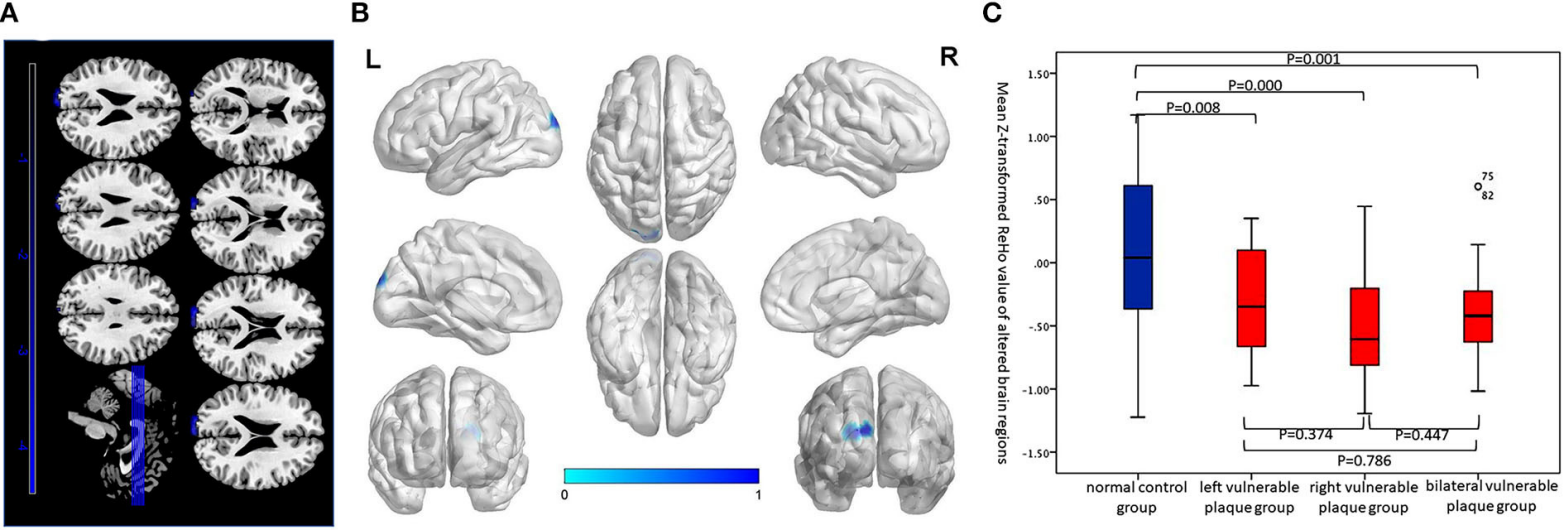
**(A,B)** Compared with the normal control group, brain regions with abnormal resting-state ReHo in the asymptomatic vulnerable plaque group, mainly located in the SOG.L (cluster size 72, *p* < 0.05, FWE correction). **(C)** Brain regions with abnormal ReHo values were extracted for multiple comparisons among the normal control group, left asymptomatic vulnerable plaque group, right asymptomatic vulnerable plaque group, and bilateral asymptomatic vulnerable plaque group. Compared with the normal control group, the left, right, and bilateral vulnerable plaque groups showed significant differences; however, the ReHo in this area did not significantly differ among the left, right, and bilateral groups.

Compared with the normal control group, the asymptomatic vulnerable plaque group showed decreased ReHo in the left superior occipital gyrus (SOG.L) (cluster size 72, *p* < 0.05, FWE correction) (Figures 1A,B and Table 2). The left, right, and bilateral vulnerable plaque groups showed no statistically significant differences in ReHo (*p* < 0.05, Bonferroni correction). In brain regions with decreased ReHo, compared with the normal control group, the ReHo in the left, right, and bilateral vulnerable plaque groups showed significant differences; however, the left, right, and bilateral vulnerable plaque groups showed no statistically significant differences in ReHo in this area (Figure 1C). No obvious relationship was found between age and ReHo (*r* = −0.167, *p* = 0.052).

**Table 2:** Brain regions with abnormal resting-state ReHo and DC in the asymptomatic vulnerable plaque group.

**Parameters**	**Brain regions**	**MNI peak coordinates**	**Cluster size**	***T*** **-value**
		**(x, y, z)**		
ReHo	SOG.L	−24, −99, 24	72.00	−4.54
DC	SOG.L	−18, −99, 24	70.00	–4.19

### DC indicator

**Figure 2:**
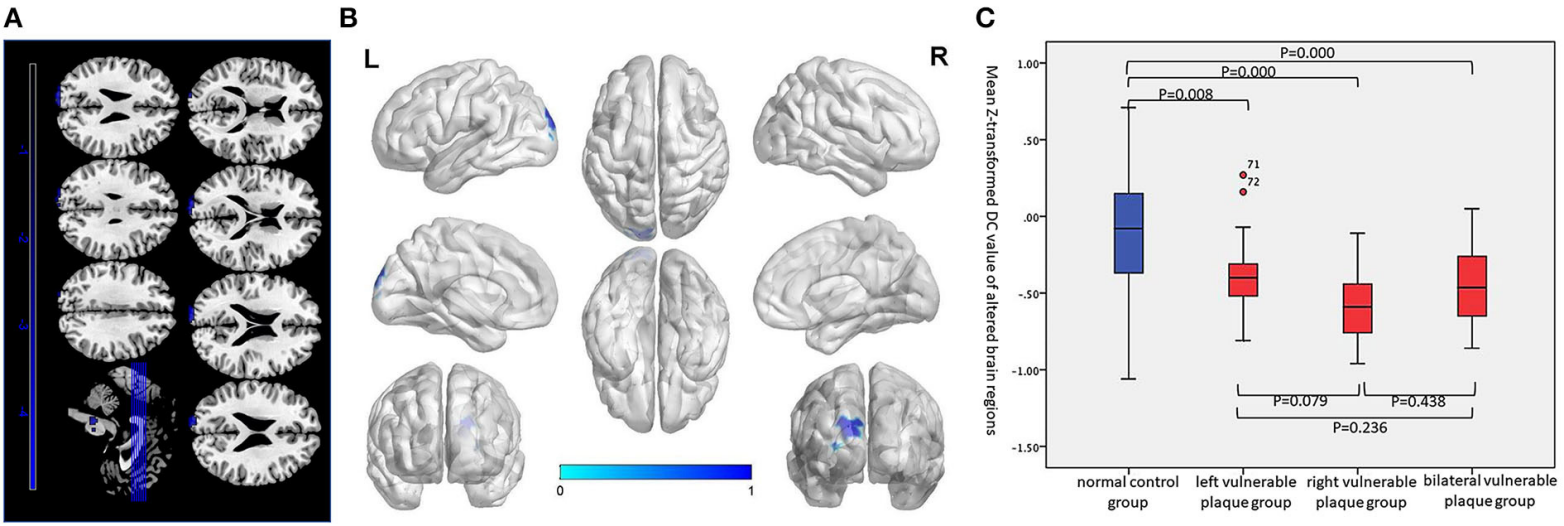
**(A,B)** Compared with the normal control group, the asymptomatic vulnerable plaque group showed decreased DC in the SOG.L (cluster size 70, *p* < 0.05, FWE correction). **(C)** Brain regions with abnormal DC values were extracted for multiple comparisons among the normal control group, left asymptomatic vulnerable plaque group, right asymptomatic vulnerable plaque group, and bilateral asymptomatic vulnerable plaque group. Compared with the normal control group, the left, right, and bilateral vulnerable plaque groups showed significant differences; however, the DC in this area did not significantly differ among the left, right, and bilateral groups.

Compared with the normal control group, the asymptomatic vulnerable plaque group showed decreased DC in the SOG.L (cluster size 70, *p* < 0.05, FWE correction) (Figures 2A,B and Table 2). The left, right, and bilateral vulnerable plaque groups showed no statistically significant differences in DC (*p* < 0.05, Bonferroni correction). In brain regions with decreased DC, compared with the normal control group, the DC in the left, right, and bilateral vulnerable plaque groups showed significant differences; however, the left, right, and bilateral vulnerable plaque groups showed no statistically significant differences in DC in this area (Figure 2C). No obvious relationship was found between age and DC (*r* = −0.065, *p* = 0.265).

### Setting brain regions with abnormal ReHo as seed points to construct voxel-wise functional connectivity networks

**Figure 3:**
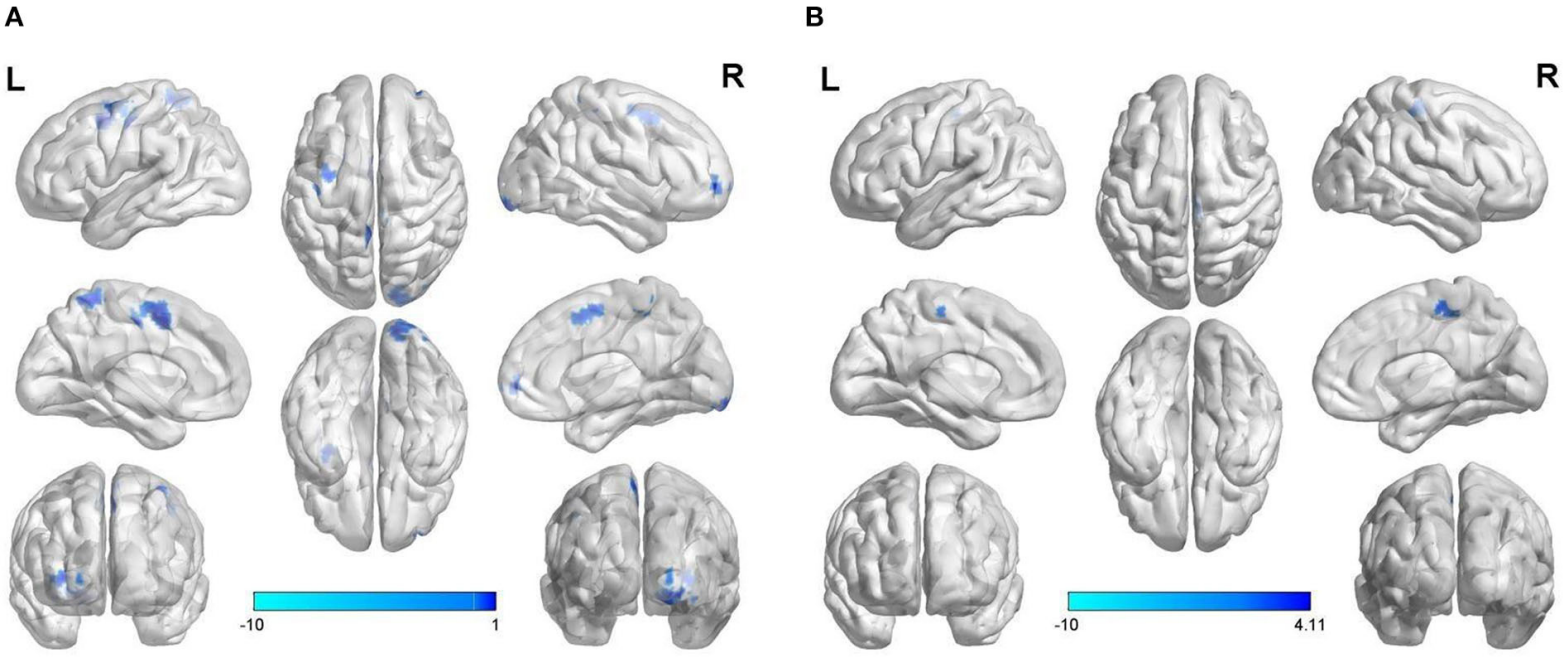
Abnormal voxel-wise functional connectivity with regions of abnormal ReHo **(A)** and DC **(B)** as seed points.

Using brain regions with abnormal ReHo as seed points to construct voxel-wise FC in the resting state, compared with the normal control group, weakened FC was found between the SOG.L and right lingual gyrus (LING.R)/right inferior occipital gyrus (IOG.R), right middle frontal gyrus (MFG.R)/orbital part of superior frontal gyrus (ORBsup.R)/orbital part of middle frontal gyrus (ORBmid.R), left precentral gyrus (PreCG.L)/postcentral gyrus (PoCG.L), PreCG.L, left supplementary motor area (SMA.L), right paracentral lobule (PCL.R), and left precuneus (PCUN.L) in the vulnerable plaque group (Figure 3A and Table 3).

**Table 3:** Abnormal voxel-wise functional connectivity with regions of abnormal ReHo and DC as seed points.

**Seed points**	**AAL brain regions**	**MNI coordinates** **(x, y, z)**	**Voxel size**	***T*** **-value**
ReHo	LING.R/IOG.R	27, –99, –12	54.00	–4.97
	MFG.R/ORBsup. R/ORBmid.R	24, 54, –9	36.00	–4.72
	PreCG.L/PoCG.L	–39, –15, 51	40.00	–4.02
	PreCG.L	–33, –6, 45	40.00	–4.65
	SMA.L	–6, 6, 57	104.00	–4.68
	PCL.R	15, –36, 54	26.00	–4.74
	PCUN.L	–3, –42, 63	18.00	–4.52
DC	PoCG.R/ PCL.R	24, –27, 54	141.00	–4.11

### Setting brain regions with abnormal DC as seed points to construct voxel-wise functional connectivity

Using brain regions with abnormal DC as seed points to construct voxel-wise FC in the resting state, compared with the normal control group, weakened FC was found between the SOG.L and right postcentral gyrus (PoCG.R)/PCL.R in vulnerable plaque group (Figure 3B and Table 3).

### Correlations of neuropsychiatric scales and abnormal ReHo and DC values

**Figure 4:**
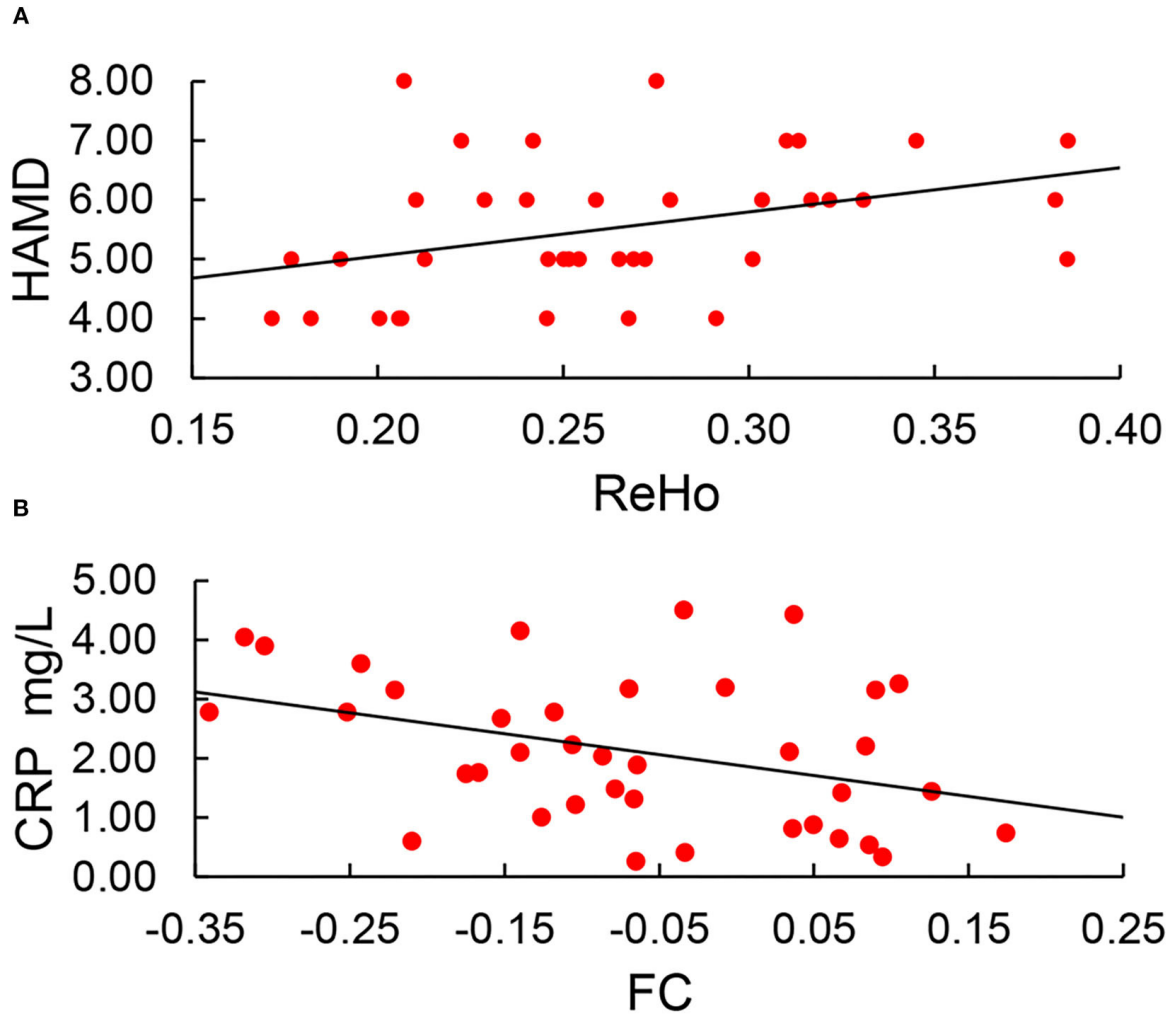
**(A)** ReHo was positively correlated with HAMD scores (*r* = 0.37, *P* = 0.022). **(B)** Setting the region of abnormal ReHo as seed points; only FC between the SOG.L and PreCG.L was negatively correlated with CRP (*r* = −0.268, *p* = 0.044).

Correlation analysis was conducted between abnormal ReHo and DC values and MMSE, HAMA, and HAMD scores. ReHo was positively correlated with HAMD scores (*r* = 0.37, *P* = 0.022) (Figure 4A). However, ReHo was irrelevant to MMSE scores (*p* = 0.122) and HAMA scores (*p* = 0.465). DC was irrelevant to MMSE scores (*p* = 0.455), HAMA scores (*p* = 0.641), and HAMD scores (*P* = 0.837). After adjusting for age and years of education, ReHo also showed positive correlations with HAMD scores (*r* = 0.37, *P* = 0.025) (uncorrected P-values are reported).

### Correlations of serum IL-6 and CRP levels with abnormal ReHo and DC values

**Figure 5:**
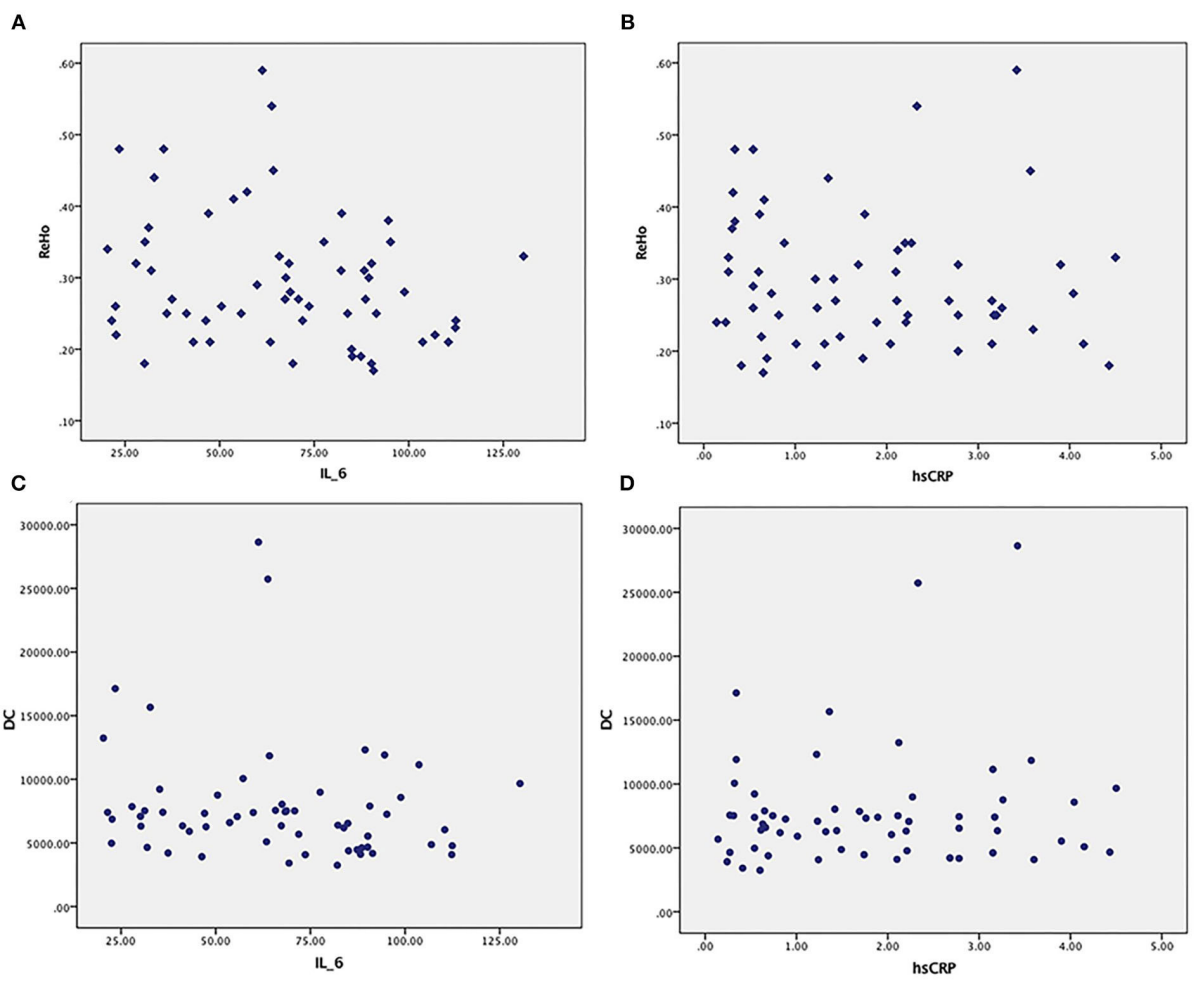
**(A,B)** No obvious relationship between ReHo in abnormal brain regions and serum IL-6 or CRP. **(C,D)** No obvious relationship between DC in abnormal brain regions and serum IL-6 or CRP.

No obvious relationship was found between ReHo in abnormal brain regions and serum IL-6 or CRP (*p* = 0.79, *p* = 0.613). No obvious relationship was found between DC in abnormal brain regions and serum IL-6 or CRP (*p* = 0.243, *p* = 0.408) (Figure 5) (uncorrected *P*-values are reported).

### Correlations of neuropsychiatric scales, serum IL-6, and CRP levels with voxel-wise abnormal functional connectivity

Setting the region of abnormal ReHo as seed points, only FC between the SOG.L and PreCG.L was negatively correlated with CRP (*r* = −0.268, *p* = 0.044) (Figure 4B). After adjusting for age and years of education, FC between the SOG.L and PreCG.L also showed negative correlations with CRP (*r* = −0.372, *p* = 0.025). No other obvious relationship was found between voxel-wise abnormal FC and neuropsychiatric scales, serum IL-6, and CRP (uncorrected *P*-values are reported).

## Discussion

In this study, we applied ReHo and DC methods to explore alterations in the resting-state brain function in asymptomatic individuals with vulnerable plaques but carotid stenosis smaller than 50% from the perspective of brain neuronal synchronization, activity, and connectivity properties. The SOG.L of the asymptomatic vulnerable carotid plaque group displayed reduced DC and ReHo value, regardless of the location of carotid plaques (left, right, or bilateral). Functional connections weakened between the SOG.L and some other brain regions, mainly the motor cortex (precentral gyrus, supplementary motor areas), the visual association cortices (occipital lobe), the somatosensory association cortex (postcentral gyrus, paracentral lobule), and the default-mode network (medial prefrontal, precuneus). In ReHo-altered brain regions, ReHo values were positively correlated with the Hamilton Rating Scale for Depression (HAMD) scores, and setting the region of abnormal ReHo as seed points, voxel-wise FC between the SOG.L and PreCG.L was negatively correlated with CRP.

The present study showed both ReHo and DC values decreased in the SOG.L in the vulnerable carotid plaque group. Some studies have discussed that alterations in the different indicators of resting-state functional MRI in the same brain regions at the same time would confirm the incident that these brain regions have changed in specific disease conditions and are robust in reliability (Martino et al., 2013; Yang et al., 2015). Thus, we can infer that the SOG.L is indeed a node with abnormal brain function in the asymptomatic vulnerable plaque group in general.

Spontaneous brain activity in the SOG.L is proved to be associated with vision (Shi et al., 2013; Huang et al., 2021) and cognitive function (Li et al., 2019; Zeng et al., 2019). Compared to the normal controls, patients with chronic stroke exhibit a significant increase in small-worldness, connection strength, betweenness centrality, and vulnerability at the SOG.L (Shi et al., 2013). The diabetic retinopathy group showed decreased functional connectivity within the bilateral lingual gyrus and from the bilateral lingual gyrus to the SOG.L (Huang et al., 2021). In addition to the visual function, previous studies have confirmed that the SOG.L is associated with cognitive impairment (Zeng et al., 2019). After meta-analysis, Li et al. (2019) found that in large-scale spatial tasks, the parahippocampal gyrus, left lingual gyrus, thalamus, right middle temporal gyrus, SOG.L, and right lenticular nucleus are highly activated, suggesting that the SOG.L is related to spatial ability, which is a core cognitive function and plays an important role in individual intelligence. Abnormalities of spontaneous brain activity or network properties in the SOG.L occurred in people with vulnerable carotid plaques in this study, and patients with diabetes and chronic stroke in previous studies, indicating that vulnerable carotid plaques and diabetes (both are stroke risk factors), together with stroke, could cause similar changes in brain activity or network properties in specific brain regions (like SOG.L) and lead to vision and cognitive dysfunctions, which need further large sample studies and cross-validation studies.

Depression is highly prevalent and persistent among survivors of stroke (Dong et al., 2022). However, the reason why patients after a stroke episode is more vulnerable to depression is still unknown. First-episode depression patients showed stronger FC in the left thalamus and reduced FC between the SOG.L and left dorsolateral prefrontal cortex seed region (Zhang et al., 2022), which suggest that abnormal connectivity of these functional circuits may be the underlying mechanism of mood disorders in patients with major depression disorder. In this study, we found that the ReHo value in the SOG.L region had altered and was positively associated with HAMD scores, indicating that the SOG.L might be a relevant node of aberrant neural networks in depression, as well as an early involved specific brain region with altered spontaneous brain activity in the vulnerable carotid plaque group. Therefore, we speculated that asymptomatic patients with vulnerable carotid plaques have a risk of depression caused by a disturbance of neuronal function in local brain regions, although these need subsequent longitudinal follow-up results.

We selected the brain regions with altered ReHo or DC values (SOG.L) as the seed points to investigate the resting-state FC of the brain. We observed weakened functional connections between the SOG.L, a site with altered ReHo value, and several other regions—the LING.R/ IOG.R, MFG.R/ORBsup.R/ORBmid.R, PreCG.L/PoCG.L, PreCG.L, SMA.L, PCL.R, and PCUN.L. A voxel-wise FC map with the sites having altered DC value as the seed points showed weakened connectivity between the SOG.L and the PoCG.R/PCL.R. First, FC was weakened between the SOG.L and some hub nodes in the default-mode network (e.g., precuneus and middle frontal gyri).The precuneus is responsible for the integration of sensory information, such as haptic and visuospatial information processing (Gentile et al., 2011). The middle frontal gyrus is a part of the medial prefrontal cortex, and the latter is a particularly important node in default-mode network. Weakened functional connections between the SOG.L and the MFG.R, as well as the PCUN.L, may lower the transmission of information between this network and other networks and affect the entire function of the default-mode network, whose function is considered an important indicator of cognitive ability (Li and Shu, 2014). Second, abnormally weakened functional connections were between the SOG.L and some other brain regions (e.g., orbital frontal gyrus and lingual gyrus) related to cognitive function. Orbital frontal gyrus abnormalities in patients with mild cognitive impairment (MCI) and Alzheimer's disease (AD) have frequently been reported in previous studies (Hoesen et al., 2000; Vogelaere et al., 2012). Also, the orbitofrontal gyrus is closely related to olfaction, and hyposmia is one of the clinical manifestations of AD (Wesson et al., 2010; Marin et al., 2018; Bathini et al., 2019; Jung et al., 2019). Also, it has been reported that abnormal lingual gyrus function is associated with impaired working memory in MCI patients (Xu et al., 2020). Last, functional connections were weakened between the left superior occipital and multiple brain regions of the motor cortex (precentral gyrus, supplementary motor areas) possibly because the SOG.L is a component of the visual cortex, and when vision is compromised, the function of the corresponding motor cortex also deteriorates.

Moreover, the left, right, and bilateral vulnerable carotid plaque groups showed no statistically significant differences in ReHo and DC values in the SOG.L region, which meant that the alterations of spontaneous brain activity in the specific brain region were independent of the laterality of vulnerable carotid plaques. Carotid atherosclerosis is considered a source of microemboli or a cause of flow-limiting stenosis and leads to ischemia or silent brain infarction (Appelman et al., 2008; Markus et al., 2010; Moroni et al., 2016), which is related to the side of the carotid plaques; however, carotid atherosclerosis could contribute to white matter lesions and ultimately lead to brain atrophy (Appelman et al., 2008; Markus et al., 2010; Moroni et al., 2016), both of which are irrelevant to side of the carotid plaque location.

Some other reasons might explain why cerebral functional changes related to the asymptomatic vulnerable carotid plaques were independent of the laterality of plaques. Considering vulnerable plaques are atheromatous plaques with possible active inflammation and atherosclerosis is proved to be a chronic inflammatory disease, we explored the relationship of the ReHo and DC values in SOG.L region as well as voxel-wise abnormal FC and serum IL-6 and CRP, which are commonly used systematic inflammatory indicators in clinics. Although our study found no correlation between serum IL-6 or CRP and the parameters of brain regions with altered ReHo and DC values, voxel-wise FC between the SOG.L and PreCG.L was negatively correlated with CRP when setting the region of abnormal ReHo as seed points. Resting-state functional connectivity is an important tool for quantifying the activity of neural circuits and showed abnormalities in some disease related to inflammation or increased serum inflammatory factors. Peripheral inflammation is associated with micro-structural changes and decreased functional connectivity in depression-related brain networks (Felger et al., 2016; Kitzbichler et al., 2021). Higher peripheral inflammatory signaling is associated with lower resting-state functional brain connectivity in emotion regulation and central executive networks (Nusslock et al., 2019). These previous studies gave us a hint that CRP may partially reflect the inflammatory process of vulnerable plaques in the carotid artery, and the potential pathological mechanisms causing decreased resting-state functional connectivity between the SOG.L and PreCG.L in patients with vulnerable carotid plaques without causing hemodynamic changes might be inflammatory.

This study had some limitations. First, the sample size was small in terms of statistical power, and further studies should enlarge the sample size. Also, the age range was very wide, although we conducted linear regression with age and ReHo, and age and DC, separately, and the statistical results turned out to have no obvious relationship; a replication of the analyses using a narrower age band would help strengthen the present results. Second, this study only performed resting-state brain function and not combining these results with structural imaging (e.g., to measure cortical thickness) and diffusion tensor imaging. Subsequent studies should use multiple imaging modalities to further verify the reproducibility and reliability of the current results. Furthermore, this study did not explore whether individuals with hard plaques and those with vulnerable plaques—the two are thought to be different in pathology—would show differences in resting-state brain function. Subsequent studies should include individuals with carotid hard plaques to further explore potential differences in resting-state brain function of the different types of carotid plaques. Last, because of lack of follow-up data, we could not draw cause-and-effect conclusions. The cognitive function and movement-related daily activities should be followed up and analyzed in the subsequent study.

In conclusion, the SOG.L was a node for alterations of neuronal synchronization, activity, and connectivity properties in asymptomatic patients with vulnerable carotid plaques but carotid stenosis smaller than 50%, confirmed by both ReHo and DC analysis, which were independent of the laterality of vulnerable carotid plaques (left, right, or bilateral). Weakened FC between the SOG.L and the motor cortex (precentral gyrus, supplementary motor areas), the visual association cortices (occipital lobe), the somatosensory association cortex (postcentral gyrus, paracentral lobule), and some nodes of the default-mode network (medial prefrontal, precuneus), and significant relation between ReHo values on the SOG.L and HAMD, both indicated that even when patients with vulnerable plaques are asymptomatic, their brain function may have changed already at the early stage of carotid atherosclerosis, and they are at risk for symptoms such as visuospatial attention decline, memory decline, and emotional dysfunction (such as depression) in future. This possibility merits further longitudinal research and provides direction regarding future possible symptoms in asymptomatic patients with vulnerable carotid plaque. Functional changes related to the asymptomatic vulnerable carotid plaques were independent of the laterality of plaques also need digging deep.

## Data availability statement

The raw data supporting the conclusions of this article will be made available by the authors, without undue reservation.

## Ethics statement

The studies involving human participants were reviewed and approved by the Ethics Committee of Xiangya School of Medicine, Central South University. The patients/participants provided their written informed consent to participate in this study. Written informed consent was obtained from the individual(s) for the publication of any potentially identifiable images or data included in this article.

## Author contributions

HJ and QW acquired data. LL provided technical guidance. LO was responsible for data analysis. QW wrote the manuscript. WX and SY revised and modified the manuscript. SY submitted the manuscript. All authors contributed to the article and approved the submitted version.

## Conflict of interest

The authors declare that the research was conducted in the absence of any commercial or financial relationships that could be construed as a potential conflict of interest.

## Publisher's note

All claims expressed in this article are solely those of the authors and do not necessarily represent those of their affiliated organizations, or those of the publisher, the editors and the reviewers. Any product that may be evaluated in this article, or claim that may be made by its manufacturer, is not guaranteed or endorsed by the publisher.
